# Conversion hip arthroplasty for failed internal fixation of ipsilateral femoral neck and shaft fractures: a case report

**DOI:** 10.1186/s13037-023-00352-1

**Published:** 2023-01-19

**Authors:** Marlon M. Mencia, Pablo Pedro Hernandez Cruz

**Affiliations:** 1 Department of Clinical Surgical Sciences, The University of the West Indies, St. Augustine, West Indies Trinidad; 2 Department of Surgery, Port-of-Spain General Hospital, Port of Spain, West Indies Trinidad

**Keywords:** Conversion total hip arthroplasty, Hip fracture, Femoral shaft fracture, Workflow, Dynamic hip screw, Retrograde femoral nail

## Abstract

**Background:**

Ipsilateral femoral neck and hip fractures are uncommon high energy injuries. In the literature no single method of treatment has emerged as superior to the others. A recent publication has documented the successful application of the rendezvous technique using dual-implants for treating these injuries. However in some cases, this technique may fail and revision surgery is required.

**Case presentation:**

A 67-year old man sustained ipsilateral fractures of his femur and femoral neck in a road traffic accident. His injuries were treated by a dual construct consisting of a retrograde femoral nail and dynamic hip screw. Three months after surgery the hip screw cut out of the femoral head necessitating revision to a total hip arthroplasty. Surgery was carried out using a single stage two part procedure on a standard operating table without having to reposition or redrape the patient. There were no postoperative complications and at 1 year from surgery the patient is satisfied with the result and has returned to work.

**Conclusion:**

Conversion hip arthroplasty in the presence of dual implants is a technically challenging and unpredictable procedure, with an increased risk of complications. Our surgical approach provides a framework for orthopedic surgeons to safely perform this complex procedure.

## Background

Ipsilateral femoral neck and shaft fractures, which are considered high-energy injuries, are thought to occur more often than previously believed [1–3]. A recent study estimates that up to 6 % of femoral shaft fractures are associated with an ipsilateral femoral neck fracture [4]. Increased awareness together with standardised diagnostic imaging protocols, including computed tomography (CT) and limited-sequence magnetic resonance imaging (MR) can help prevent missed femoral neck fractures which may occur in 30% of these injuries [3, 5–7]. Over the last decade, several surgical options have been proposed to manage these injuries but no single procedure has been shown to be superior to the others [8–10]. More recently, dual-implants consisting of a dynamic hip screw (DHS) and a separate retrograde femoral nail have been shown to result in fewer re-operations [11, 12]. Using this technique, Ostrum et al. reported that 98% of femoral neck fractures healed uneventfully [4].

In our recent report, we described the dual-implant rendezvous technique as an effective treatment for ipsilateral femoral neck and shaft fractures [13]. While this technique often produces good results, there are occasional instances where the femoral neck fixation fails, requiring revision surgery [14, 15]. In these cases, conversion total hip arthroplasty is the preferred option for revision [14, 15].

Conversion total hip arthroplasty can be a complex and risky procedure when only a single implant is present [16–22]. To reduce complications it is critical for surgeons to carefully plan the procedure and follow a precise surgical technique [21, 22]. This is especially true in low-resource environments where access to specialized equipment and expertise may be limited. To date, there are no published reports on the use of conversion total hip arthroplasty in cases where dual implants are present. In this manuscript, we present a logical surgical approach for managing these challenging cases.

## Case presentation

**Fig. 1:**
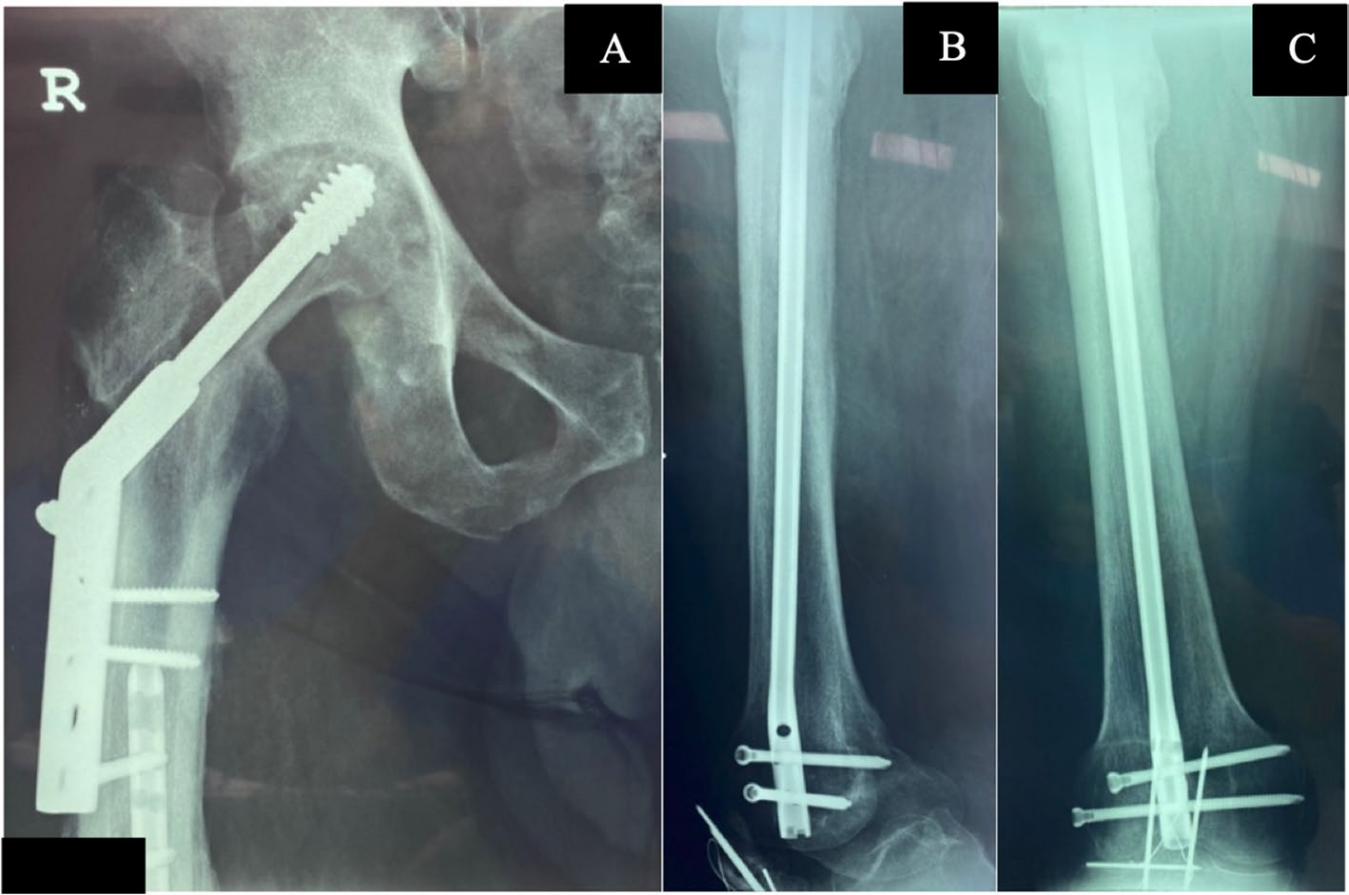
Anteroposterior radiograph of the hip (**A**) shows cut out of the lag screw in the DHS. Complete union of the associated femoral shaft fracture in both the lateral (**B**) and anteroposterior (**C**) radiographs with the retrograde femoral nail in-situ

A 67-year-old man was involved in a motor vehicle accident and sustained a comminuted mid-shaft fracture of his femur and ipsilateral patella. After being admitted to the hospital, his injuries were treated using a retrograde femoral nail and tension band wiring of the patella. However, postoperative radiographs revealed a previously undiagnosed fracture of the femoral neck, which was subsequently stabilized with a four-hole 135° dynamic hip screw (DHS). Three months later, radiographs showed that the DHS had cut out of the femoral head while both the femoral shaft and patella fracture were solidly united. Figure 1A, B & C. Clinically, the patient was in severe pain with limited knee movement (10°-90°) and a preoperative EuroQol-5 D (EQ-5D) and EuroQol-Visual Analogue Scores (EQ-VAS) scores of 0.312 and 20, respectively. To address this issue we advised the patient to undergo conversion total hip arthroplasty. The patient gave informed consent for his anonymized information and photographs to be used in the preparation of this case report.

Before surgery we conducted a comprehensive preoperative workup to exclude infection and carefully examined the patient’s recent radiographs to identify failed implants, particularly broken screws or the intramedullary nail which can present additional challenges to remove.

We performed the operation as a single-stage, two-part procedure. The first part involved removing all implants and thorough debridement of the bone and soft tissues. The second part involved the total hip arthroplasty. Both parts were carefully choreographed so that there was no need to re-prep or change the patient’s position during surgery. This created a smooth, coordinated workflow in the operating theatre.

The patient received a spinal anesthetic and was placed in the lateral decubitus position. We cleaned and prepared the limb for surgery, allowing access from the hip to the mid-leg. Prophylactic antibiotics and tranexamic acid were administered. The surgical technique consisted of a series of logical, well-coordinated steps.

First, we removed the distal locking screws from the retrograde femoral nail. To prevent damaging the hex of the screw head, we made sure that the screwdriver was firmly engaged in the hex. This is important to avoid iatrogenic stripping, which can complicate the removal process.

**Fig. 2:**
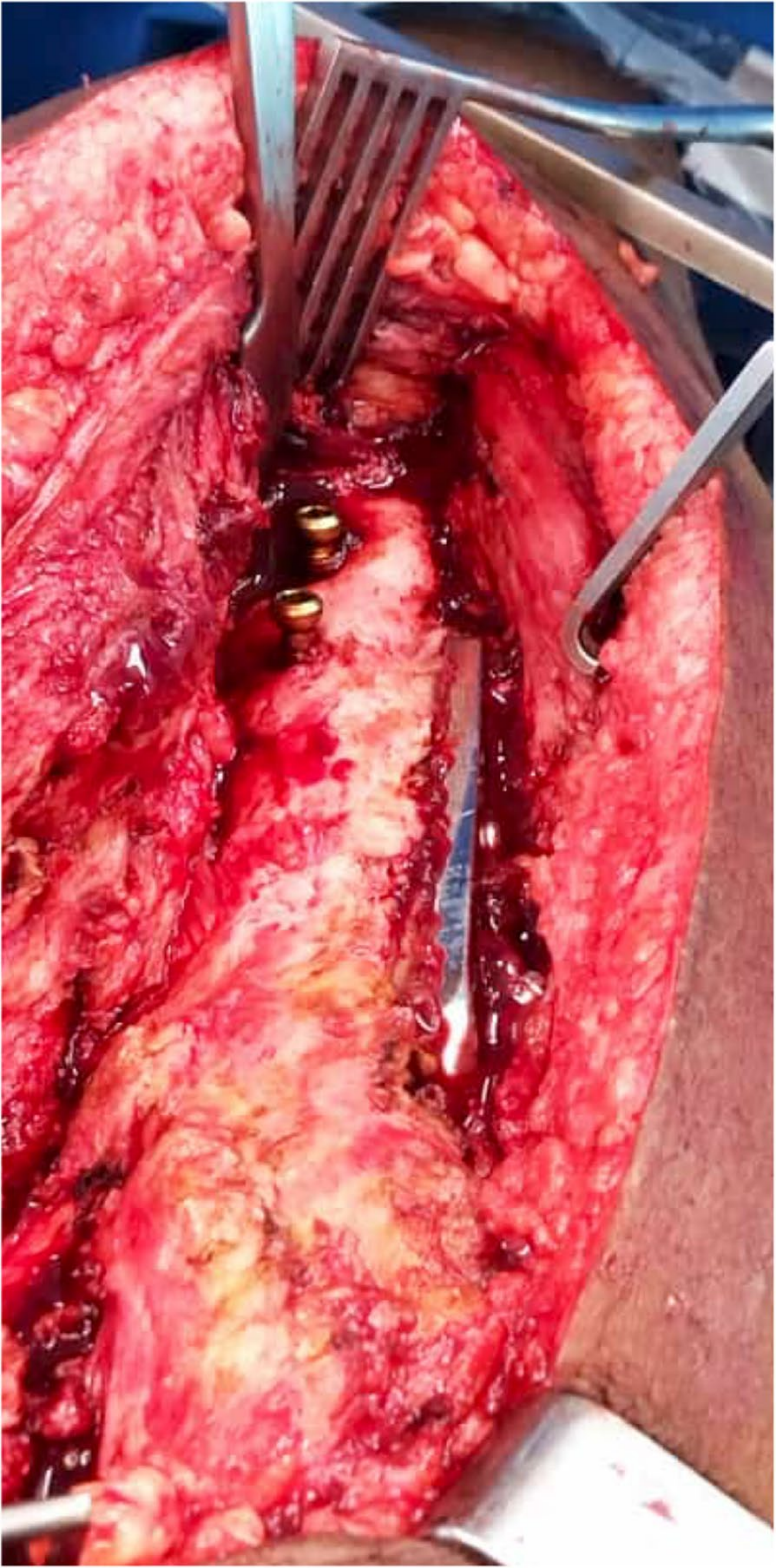
Clinical photograph shows the side plate of the DHS and the anteroposterior locking bolts of the retrograde femoral nail under the anterior fibres of vastus lateralis

Second, we directed our attention to the hip; using a modified Hardinge approach, our incision began 8 cm proximal to the tip of the greater trochanter and extended down the line of the femur incorporating the surgical scar. A generous incision permits good visualization. Our deep dissection was carried out by separating the fibres of gluteus medius longitudinally then extending distally into vastus lateralis, where the DHS side plate was identified and exposed. After releasing all adhesions we were able to easily dislocate the hip by gentle external rotation. The trauma implants were then removed, including the proximal locking bolts of the retrograde femoral nail, which are readily accessed under the anterior part of vastus lateralis Fig. 2.

**Fig. 3:**
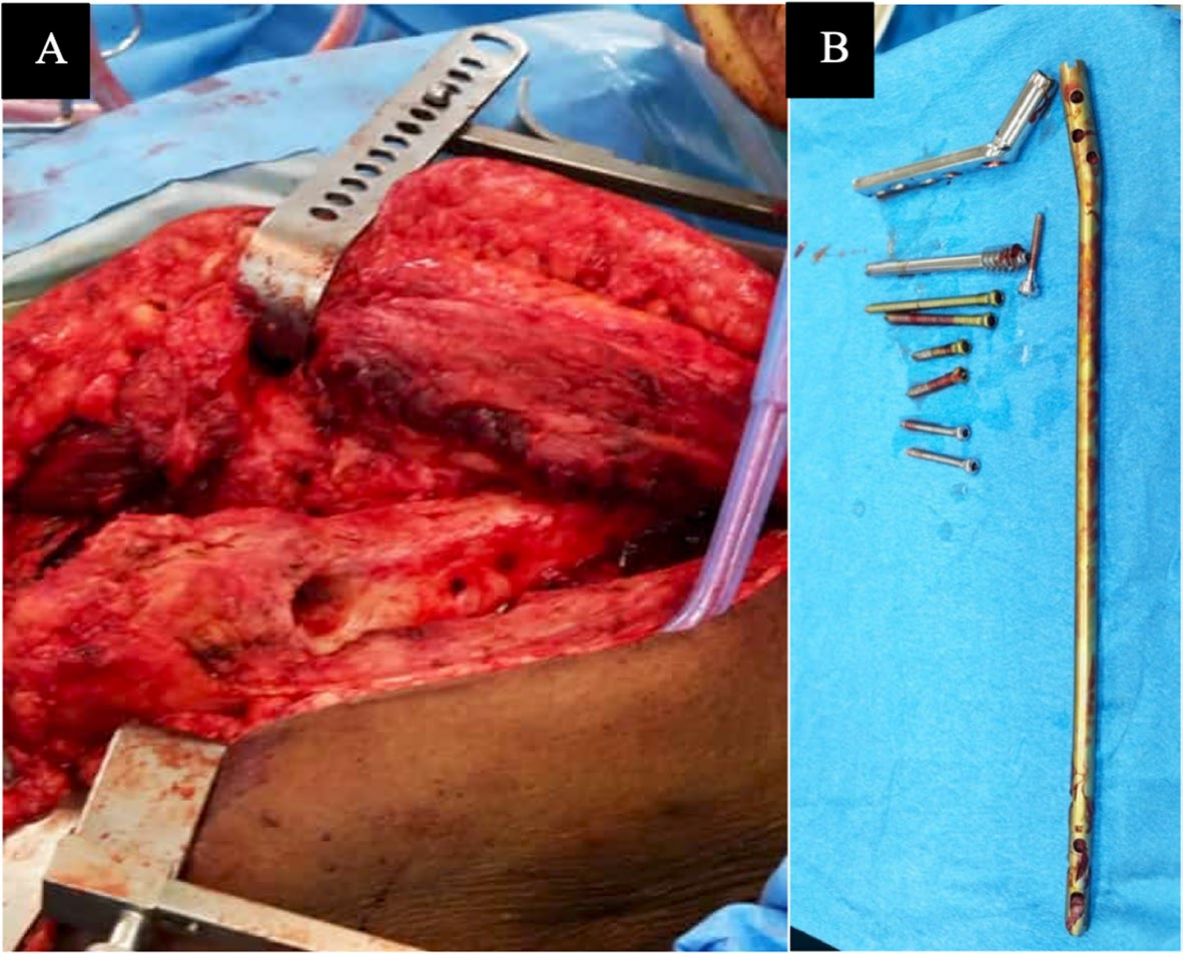
Clinical photograph (**A**) showing the residual screw holes left after removal of implants. All implants removed before proceeding to total hip arthroplasty (**B**)

Third, with the hip dislocated, we covered the soft tissues with a moist lap sponge and flexed the knee to approximately 30°. Using a midline incision, we split the patella tendon and excised any remaining fat pad or fibrous tissue to access the femoral notch. We located the end of the nail, which was then easily removed using the extraction device, and the knee joint was irrigated before closing the skin Fig. 3.

**Fig. 4:**
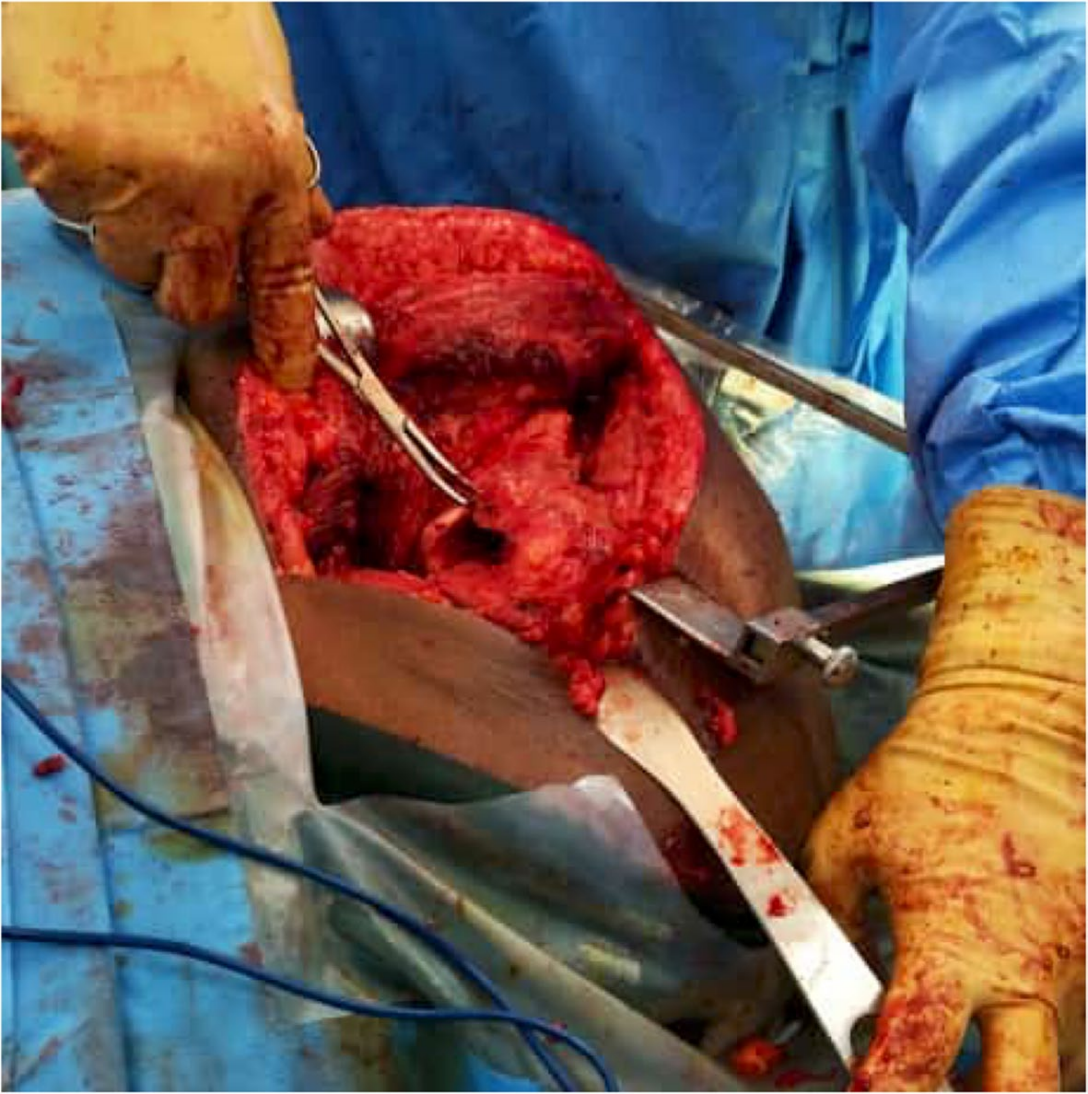
Adequate exposure of the entry point allows for safe preparation of the femoral stem

**Fig. 5:**
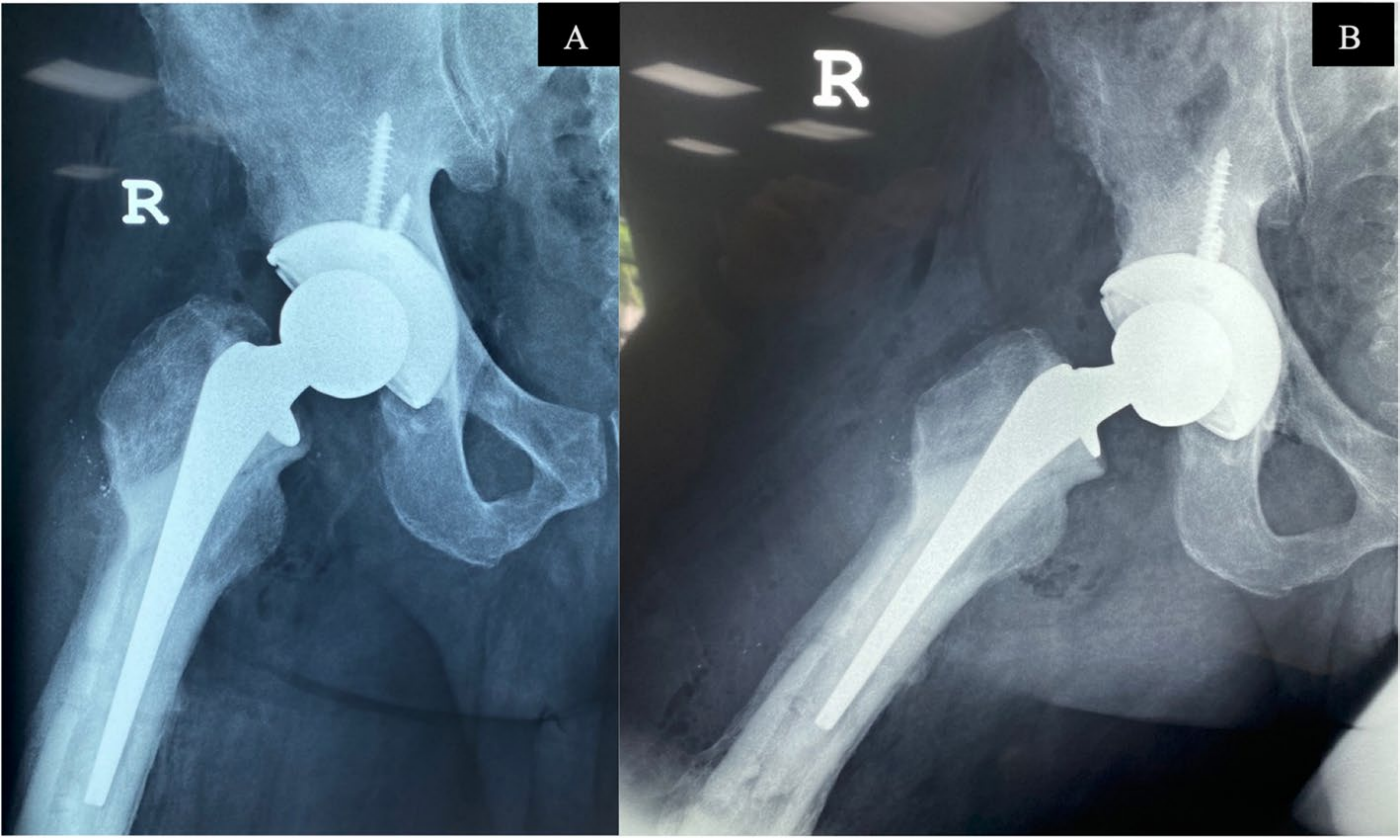
Post-operative anteroposterior (**A**) and lateral radiographs (**B**) after removal of all implants and revision to total hip arthroplasty

Finally, we performed a standard hip arthroplasty with a few key technical considerations. First, the acetabular bed must be carefully prepared to support an uncemented acetabular shell, as the bone may have become soft. We used two screws to enhance the primary stability of the implant. As the surgery progresses, it is important to maintain good exposure and visualization of the femoral entry point to prevent iatrogenic fracture of the malunited greater trochanter Fig. 4. After creating the entry point, we used a long drill bit to find the center of the femoral canal. This is crucial, as intramedullary bone bridges may block or misdirect the hip reamers, leading to perforation or fracture of the femur. We then exchanged the drill bit for a guidewire and used flexible reamers to enlarge the femoral canal. Trial components were used to assess hip stability and leg length and we cemented the femoral stem using a third-generation cementation technique. Our operating time was 127 mins with an estimated blood loss of 400mls. The patient was able to bear full weight the day after surgery and was discharged on the third day Fig. 5A & B. One year after surgery the patient was walking unassisted and has returned to work as a mason. The postoperative Oxford Hip Score, EQ-5D, EQ-VAS are 43, 0.864 and 84 respectively.

## Discussion

Conversion THA is a complex surgical procedure, particularly when multiple implants need to be removed. A well-organized surgical workflow can reduce the risk of surgical errors, minimise operating time, lower costs and improve clinical outcomes. One study found that disruptions in the surgical workflow accounted for up to 20.5% of operating time, highlighting the importance of surgeons paying attention to their workflow during the procedure [23].

“If you fail to plan, you are planning to fail” is a well-known quotation from Benjamin Franklin, a renowned American polymath and one of the leading intellectuals of his time.

The quote is particularly relevant to complex surgical procedures where errors may result in death. If used correctly preoperative planning can improve surgical outcomes and prevent unanticipated problems. Surgical tactic which is one of the three main elements of surgical planning is attributed to Maurice Müller who described a logical and progressive step by step guide to surgery [24, 25]. Surgical tactic can be thought of as a type of workflow in which an operation proceeds smoothly with an economy of movement, efficient use of time and a successful outcome. Surgical workflow has important clinical implications and Wiegmann et al. have shown that even minor disruptions can result in surgical errors and complications [26]. The problem is exaggerated in complex tasks and in particular uncommon procedures, which is typical of conversion total hip arthroplasty as described earlier.

Selecting an appropriate femoral stem is an important decision point in conversion total hip arthroplasty that can help to reduce the complications. Avoiding a periprosthetic fracture should be the surgeon’s main priority. Periprosthetic fractures are a constant hazard during conversion total hip arthroplasty where they occur more commonly than during primary THA. While these fractures may occur during the intra or post-operative period, most studies report combined periprosthetic fracture rates, which can be misleading because the causes of intraoperative and postoperative fractures are different. For example patients treated by a DHS are known to have low bone quality secondary to stress-shielding, which increases the risk of a late postoperative femoral fracture [27]. On the other hand, intraoperative femoral fractures are more technique dependent and are therefore preventable.

A much debated question in conversion total hip arthroplasty is the method of stem fixation. Some surgeons argue that cementless stems, which rely on a tight implant-bone fit to provide primary stability and osseointegration, increase the risk of intraoperative fractures compared with cemented stems.

The reported rate of periprosthetic fractures following conversion total hip arthroplasty ranges widely from 0 to 39% [15, 17, 27, 28]. Many of these studies are small (< 50 patients) so it is difficult to draw firm conclusions. In the two largest studies each with over 100 patients and using several different stems-types, identical intraoperative fractures rates of 4% were reported [15, 17]. In the study by Mortazavi et al., 21% of intertrochanteric fractures required prophylactic cerclage wiring during conversion total hip arthroplasty using cementless stems, which may prevented an intraoperative fracture and artificially lowered the fracture rate [17]. Similarly, Archibeck et al. reported four periprosthetic fractures at the level of the cortical screw hole all occurring in patients with prior intertrochanteric fractures. Of these, three occurred with an uncemented stem and one with a long-stem cemented prosthesis [15]. The higher risk of diaphyseal periprosthetic fractures in this group of patients prompted the authors to recommend routine use of prophylactic cerclage wiring in agreement with Mortazavi et al. [17]. We are therefore left to conclude that existing studies so far provide conflicting evidence concerning the most appropriate choice of femoral stem. In our case, the decision to use a cemented stem was influenced by weak proximal bone stock which together with several multiplanar cortical screw holes would have increased the risk of a femoral fracture.

## Conclusion

Conversion THA is a technically demanding surgical procedure with a higher complication rate than primary arthroplasty. When dual implants are used, conversion total hip arthroplasty becomes even more complex, and surgeons should be aware of its peculiar challenges. However, the rarity of this scenario means that, except in specialized centers, surgeons have relatively little experience in treating such cases. In developing countries with less sophisticated health systems, general orthopedic surgeons, often with limited resources, are often the ones who are called upon to treat these patients. As a result, it is not surprising that conversion total hip arthroplasty is associated with high complication rates. When confronted with unfamiliar and complex surgical procedures, surgeons must approach problem-solving in a thoughtful manner that is consistent with their working environment. Our surgical tactic and choice of stem provide orthopedic surgeons with a step-by-step method for complex hip reconstruction that prioritises patient safety and minimizes the risk of complications.
